# [Retracted] Prognostic significance of miR-218 in human hepatocellular carcinoma and its role in cell growth

**DOI:** 10.3892/or.2026.9051

**Published:** 2026-01-19

**Authors:** Kangsheng Tu, Chao Li, Xin Zheng, Wei Yang, Yingmin Yao, Qingguang Liu

Oncol Rep 32: 1571–1577, 2024; DOI: 10.3892/or.2014.3386

Following the publication of the above paper, it was drawn to the Editor's attention by a concerned reader that certain of the flow cytometric data shown in Fig. 3C, the immunohistochemical data shown in Fig. 4B and the western blots in Fig. 5B had already been submitted to, or were published in, articles in other journals that featured some of the same authors; moreover, some of these data subsequently appeared in different articles in other journals that were not connected with either this research group or this research topic. Upon investigating these issues further in the Editorial Office, it was noted that, concerning Fig. 3, Fig. 4, Fig. 5 and as far as those papers sharing some of the same authors was concerned, the cases of data sharing weren't necessarily as simple as the data merely being duplicated.

Given the sharing of these contentious data across a number of different journals, the Editor of *Oncology Reports* has decided that this paper should be retracted from the Journal on account of a lack of confidence in the presented data. The authors were asked for an explanation to account for these concerns, but the Editorial Office did not receive a reply. The Editor apologizes to the readership for any inconvenience caused.

